# Instability Compensation of Recording Interferometer in Phase-Sensitive OTDR

**DOI:** 10.3390/s24113338

**Published:** 2024-05-23

**Authors:** Konstantin V. Stepanov, Andrey A. Zhirnov, Tatyana V. Gritsenko, Roman I. Khan, Kirill I. Koshelev, Cesare Svelto, Alexey B. Pnev

**Affiliations:** 1 Laser and Optoelectronic Systems Department, Radio Electronics and Laser Technology Faculty, Bauman Moscow State Technical University, 2-nd Baumanskaya 5-1, 105005 Moscow, Russia; a.zh@bmstu.ru (A.A.Z.); chobantv@yandex.ru (T.V.G.); khan.roman.igorevich@gmail.com (R.I.K.); koshelev-k@yandex.ru (K.I.K.); pniov@bmstu.ru (A.B.P.); 2 Dipartimento di Elettronica, Informazione e Bioingegneria, Politecnico di Milano, 20133 Milan, Italy; cesare.svelto@polimi.it

**Keywords:** fiber optic sensor, distributed fiber optic sensor, phase-sensitive OTDR, acoustic monitoring, weak fiber Bragg gratings (WFBG)

## Abstract

In the paper, a new method of phase measurement error suppression in a phase-sensitive optical time domain reflectometer is proposed and experimentally proved. The main causes of phase measurement errors are identified and considered, such as the influence of the recording interferometer instabilities and laser wavelength instability, which can cause inaccuracies in phase unwrapping. The use of a Mach–Zender interferometer made by 3 × 3 fiber couplers is proposed and tested to provide insensitivity to the recording interferometer and laser source instabilities. It is shown that using all three available photodetectors of the interferometer, instead of just one pair, achieves significantly better accuracy in the phase unwrapping. A novel compensation scheme for accurate phase measurements in a phase-sensitive optical time domain reflectometer is proposed, and a comparison of the measurement signals with or without such compensation is shown and discussed. The proposed method, using three photodetectors, allows for very good compensation of the phase measurement errors arising from common-mode noise from the interferometer and laser source, providing a significant improvement in signal detection. In addition, the method allows the tracking of slow temperature changes in the monitored fiber/object, which is not obtainable when using a simple low-pass filter for phase unwrapping error reduction, as is customary in several systems of this kind.

## 1. Introduction

Fiber optic sensors have been rapidly developing and attracting increasing interest since the end of the last century [1,2,3,4,5]. They have several advantages over traditional electrical sensors, such as the absence of an electric power supply, which makes them completely explosion- and fire-proof, the relative ease of multiplexing, and the large distance from the “intelligent” and active part of the system to the sensing part of the sensor, which is distributed and fully passive. In particular, the so-called distributed fiber systems are worth highlighting due to their wide availability and unique properties. In such systems, an ordinary telecommunications fiber can be used as the sensor and the exposure/sensing parameters are recorded with a certain sampling length along the entire fiber path. The whole fiber length can reach from some tens of meters up to hundreds of kilometers, with the typical optical fiber electromagnetic immunity, light weight, robustness, and low cost.

These types of sensors include distributed fiber optic sensors based on the effects of Rayleigh scattering in a fiber. Such sensors include two types of methodologies: a first one, named Optical Frequency Domain Reflectometry (OFDR), which works in the frequency domain using continuous wave light sources and then obtains the measurement data from the Fourier transform of the signal [6,7,8,9]; and a second one, commonly named Optical Time Domain Reflectometry (OTDR) from [10,11,12], where the analysis of the measurement signal is performed in the time domain. Provided that the coherence length of the probing optical pulse sent along the fiber is greater than the spatial length of the pulse itself into the fiber, from an OTDR-detected signal, it is possible to register acoustic and vibration interactions acting on different sections of the whole sensor cable: for example, footsteps, vibrations arising from moving vehicles or from a digging tool, unwanted intrusions in the proximity of the fiber, etc. More generally, these measurement systems have a sensitivity to any physical and mechanical quantity acting on the thermo-mechanical properties of the fiber under test and producing different phase shifts in the backscattered waves that are detectable by a phase-sensitive OTDR system (φ-OTDR).

The beginning of such sensing systems in the 1990s was led by a group of researchers at the University of Texas under the leadership of H.F. Taylor [13,14,15,16,17]. Over the following decades, much research has been undertaken not only in scientific achievements and understanding of the involved phenomena but also in the practical use and commercialization of measurement systems of this type [18,19,20,21,22]. A huge number of schemes and methods have been proposed and tested to increase the sensitivity of the system [23,24,25,26,27,28], the length of the sensor cable [29,30,31], and the number of registration channels [32,33]. Distributed fiber-optic systems are mostly popular for recording acoustic exposures in the field of pipeline monitoring against unauthorized intervention, as well as recording pipeline rupture points and leakage [34,35]. Remote monitoring and control of the movement and speed of trains is another common application [36,37]. Seismic profiling is also worth mentioning, where a φ-OTDR is used as an alternative to classic point sensors and allows the spatial recording of the required parameters in a single measurement [38,39,40]. Recently, the use of neural network algorithms for processing the recorded data of a φ-OTDR has been gaining popularity, not only to determine the event of exposure but also to classify it [41,42,43,44,45,46,47].

In addition to detecting the happening of an event acting on the optical fiber—for example unauthorized work in telecommunication wells [41] and in a number of other tasks, such as measuring oil well parameters [38,39,48,49,50]—it is necessary to quantitatively detect the absolute value of the phase change. This requires unwrapping the phase signal [51] and obtaining an analog-and-continuous measurement of the phase without 2π phase jumps.

**Figure 1:**
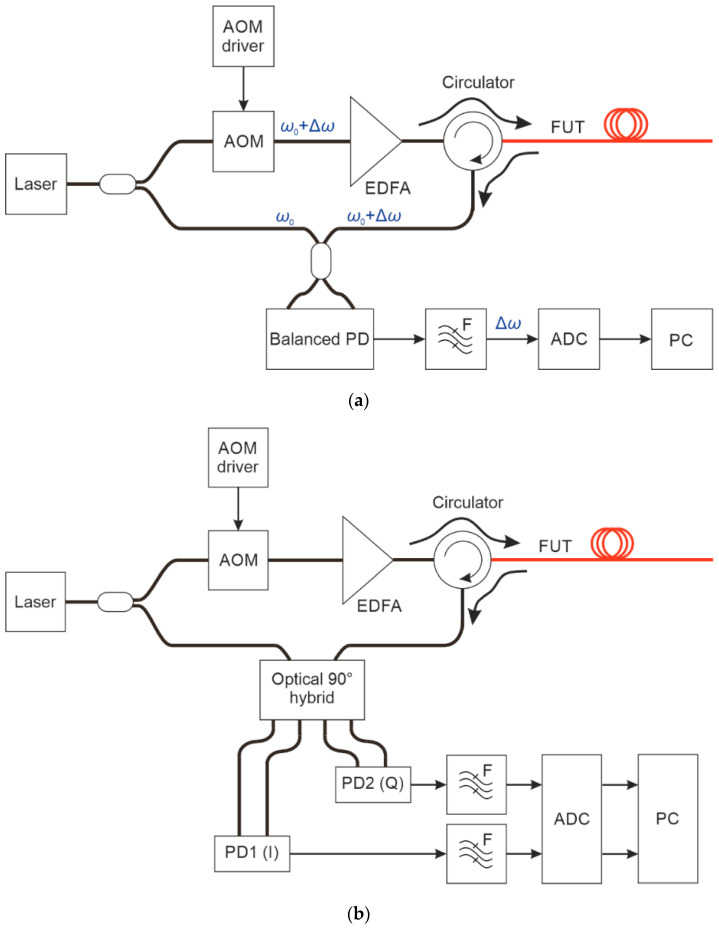
Scheme of a φ-OTDR with phase recovery (coherent): (**a**) Heterodyne, with frequency shift; (**b**) Homodyne, with baseband detection.

**Figure 2:**
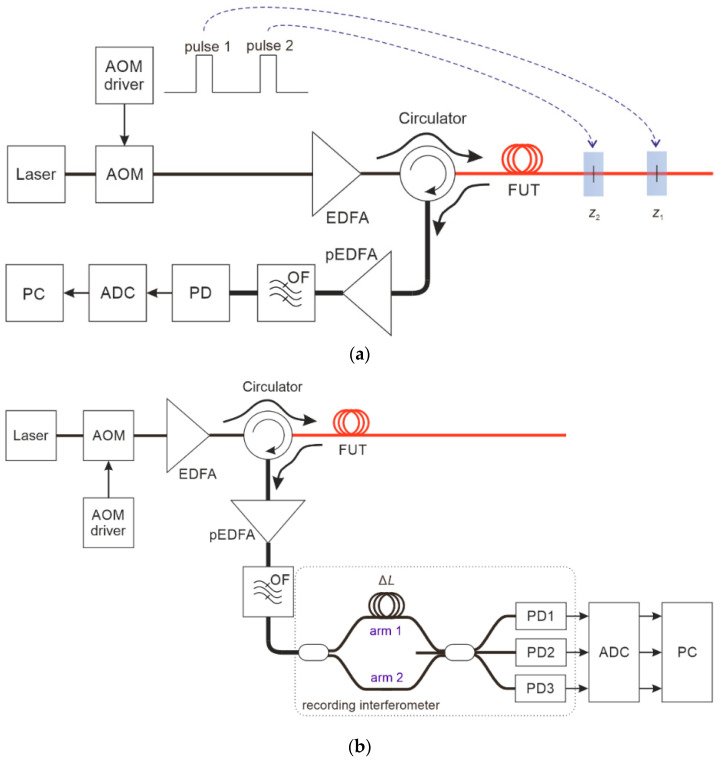
Scheme of a φ-OTDR with direct detection phase recovery: (**a**) With phase difference and time delay between pair of pulses; (**b**) With interference of two reflectograms shifted in time at the receiver.

To perform quantitative measurements using φ-OTDR, detection systems with phase-unwrapping capabilities must be used. In these systems, the sensitive element is not the section of the fiber in which there is a pulse at a particular moment in time but the section of the fiber between two positions of this pulse. The length of this section is called “gauge length”, and it does not necessarily need to be equal to the pulse half-width as it can also be longer. There are two main methods for implementing these schemes:

(i) A scheme with coherent detection that can be implemented according to two different cases: based on a heterodyne or homodyne principle. In the first case (Figure 1a), the phase of radiation for each fiber under test (FUT) coordinate is calculated as the phase of the carrier frequency generated by the offset of the acousto-optical modulator (AOM), IQ modulator, or another frequency shifter, located in the transmitting part. A balanced or coherent photodetector (PD) can be used to detect the backscattered radiation. However, the ADC sampling rate needs to be relatively high to ensure a small phase error. In the second case (Figure 1b), i.e., when using homodyne detection, the radiation in the transmitting part is only on–off amplitude modulated, and no frequency shift is applied. This allows maintaining the minimum frequency range for the signal recording (required by the pulse spectrum width), but it constrains working inside the low-frequency noise spectral region, where more noise is superimposed on the signal. Typically, such a homodyne scheme uses only one coherent receiver to record the phase of the scattered radiation wave field. The common advantage of these coherent-detection methods is the low attenuation of the signal amplitude, with a coefficient α = 0.18 dB/km along the fiber (SMF28 standard value), which allows doubling the attenuation-limited registration range as compared to the direct-detection schemes discussed below. The disadvantage is the significantly high cost of the coherent scheme components and the high-speed signal processing resources.

(ii) A scheme with direct detection that can be either implemented by sending optical pulses with a phase shift and a time delay between them in the transmitting part (Figure 2a) or by shifting the reflectograms and adding a time delay due to arms unbalancing, in the recording interferometer placed in the receiving part (Figure 2b). In this last case, the time delay in the two interferometer arms will determine the gauge length, and its value is fixed and cannot be changed by the adjustable system’s parameters when processing the signals from the receivers. However, this drawback is more than compensated by the significant advantage that all the calculations of the phase difference occur in the fiber at the physical level, and it does not require computing resources or additional electrical bandwidth. Other relative disadvantages of direct detection include a higher optical signal attenuation with a coefficient of 2 × α = 0.36 dB/km, thus reducing the useful sensor length; the requirement for an optical preamplifier (Erbium-doped fiber amplifier (EDFA) at the receiver, since there is no signal amplification by a local oscillator; and also the need either to send three pairs of pulses (reducing the frequency response and measurement speed of the φ-OTDR sensor by 3 times) or to use three receivers and a triple-ADC stage (although all of these requirements are less technologically complicated and far less expensive than the devices needed in the case of a coherent receiver).

Although they are based on simpler direct optical detection, an additional complexity of signal processing in incoherent systems is the need for a procedure of phase unwrapping the measurement signal along the time axis. This process allows for the correct unwrapping of the shape of signals whose total phase excursion exceeds ±π radians, but the procedure is not robust, and it strongly depends on the SNR and pulse repetition rate. In addition, an incorrect result of the phase unwrapping operations can lead to large signal distortions and even to the generation of false events. Therefore, in some cases, additional high-pass filtering of the detected signal is adopted, which removes slow and cumulative phase additions resulting in “jumps” in the phase signal. Usually, frequencies higher than 10 Hz contain information about the physical signal acting on the FUT, while the mean level is zeroed by excluding low-frequency perturbations. However, along with the useful filtering of the low-frequency disturbances, the high-pass filtering used in the unwrapping procedure can also remove some useful information that the fiber sensor was able to detect without this filtering. The described schemes for quantitative measurements are linear in terms of transfer characteristics but have a limited working range in frequency and amplitude (problem described in [52]). On the other hand, for the previously mentioned qualitative measurements (with only intensity detection without phase reconstruction), there are no such limitations since, with backscattered intensity detection and nonlinear transfer characteristics, the only disadvantage is that of a nonlinear transfer function.

The error in phase unwrapping of φ-OTDR systems depends on many factors, such as the distance to the point of the event or “impact” (since the signal-to-noise ratio is directly related to the distance), the quality of the radiation source stability (assessed by its relative intensity noise, RIN, and its phase noise), the photodetector noise, temperature gradients, and the overall vibration immunity of the system. One of the reasons why the last two effects are so important is that the phase unwrapping is carried out using a recording interferometer in the receiving part of the system, and the thermomechanical instability of the internal components of the interferometer makes a large contribution to the phase error. Usually, a Mach–Zehnder interferometer (MZI) is used to record the phase signal, and the path difference between the sensing and reference fiber arms of the MZI corresponds to the spatial resolution of the φ-OTDR system.

For the MZI in the receiving part, the resulting signal can be determined by the following formula:

(1) $$I\left(t\right)=Acos\left(\frac{2\pi \Delta L\left(1+k\left(T-T_{0}\right)\right)}{\lambda \left(t\right)}\right)+N\left(t\right),$$

where $I\left(t\right)$—the interference result in relative units in the range of values from −1 to 1 at a time moment *t*;

ΔL—MZI’s arms unbalance;

*k*—coefficient of linear thermal expansion of fiber;

*T* and *T*_0_—fiber temperature at the current and at the initial moment, respectively;

λ(t)—wavelength of the laser source at the current moment;

$N\left(t\right)$—noise of photodetectors and ADC.

Under such conditions, it is clear that with an arms’ difference of 20 m, a temperature change of even Δ*T*_MZI_ = 0.01 K will lead to a measurement signal phase change of 0.8 radians, which is an extremely significant value within the framework of the measurements being carried out. At the same time, the stability of the laser wavelength, with a stated linewidth of 100 Hz (less than one attometer, over some hundreds of microsecond integration time) and wavelength fluctuation (improperly called drift) of less than a few femtometers per minute give an absolutely negligible contribution to the phase measurement error. Given commonly achievable values of the laser temperature stability and a stable laser temperature of Δ*T*_LASER_ ≤ 0.05 K, the previous wavelength fluctuation resulted in a phase change of only 0.05 radians or less, which is two orders of magnitude smaller than the effect due to temperature changes in the interferometer fiber. 

Thus, the interferometer instability is the main cause of phase noise and errors in this kind of φ-OTDR phase signal. Finally, one could think of passively and actively stabilizing the interferometer, with respect to temperature fluctuations and vibrations, in order to reduce its dominant effect on the error in phase reconstruction. Unfortunately, the required temperature stabilization, at a level of 0.01 K or even less, for the recording interferometer assembly is an extremely difficult and complex task, requiring unprecedented development of the casing design.

**Figure 3:**
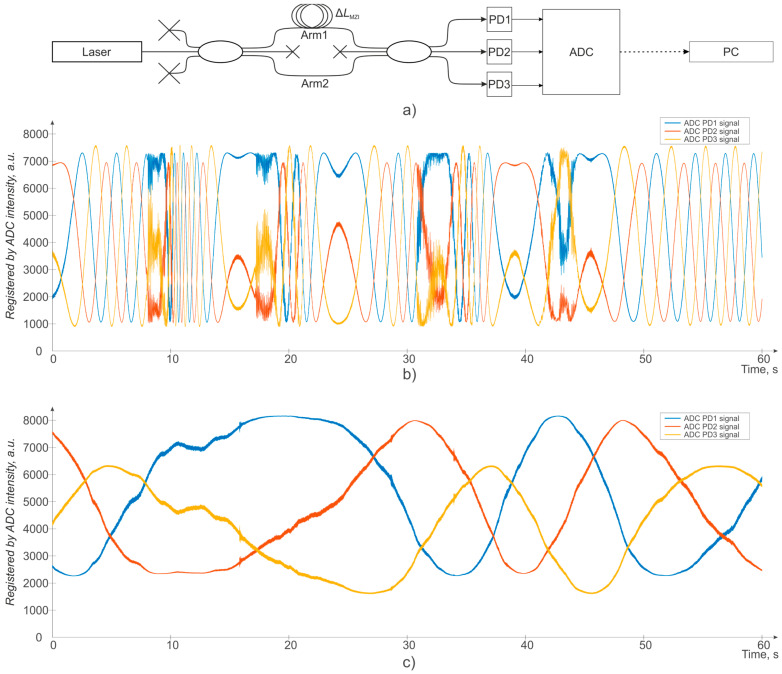
The temperature gradients and the overall vibration influences on the recording interferometer: (**a**) Registration scheme; (**b**) MZI is not placed in an isolation box; (**c**) MZI is placed in a special cumbersome isolation box.

An example of a specific implementation of the temperature gradients and the overall vibration influences on the recording interferometer within 60 s of observation time is shown in Figure 3. The simplest recording scheme for instability registration of the recording interferometer is shown in Figure 3a: a highly stable laser with a bandwidth of 100 Hz was used as a radiation source to maximize and exclude its contribution to the error. The measurement results when the MZI is not placed inside an isolation box, with the interferometer affected by both temperature drifts and vibrations and air flows, for example, from case fans, are shown in Figure 3b. The results when the MZI is placed in a special cumbersome insulated box, vibrationally decoupled from itself, including fans, are shown in Figure 3c. It is worth noting that the insulated box used was similar in size to a full-size Φ-OTDR device.

As can be seen from Figure 3b,c, the temperature and vibrations’ influence on the recording interferometer is significant, resulting in large variations of the interferometric signal, and it can severely limit the applicability of such φ-OTDR. To solve this problem, a more detailed study of this known dependence should be conducted, and other ways to physically and directly compensate for the instability of the φ-OTDR recording interferometer should be developed, which will provide for more accurate and stable phase recovery with φ-OTDR, but this is beyond the scope of this work.

This paper proposes innovative ways for the compensation of the instability of the φ-OTDR recording interferometer, acting indirectly on the different available interferometer signals, and also presents, both theoretically and experimentally, a comparison of detected unwrapped-phase signals with or without such compensation methods.

## 2. Theory

**Figure 4:**
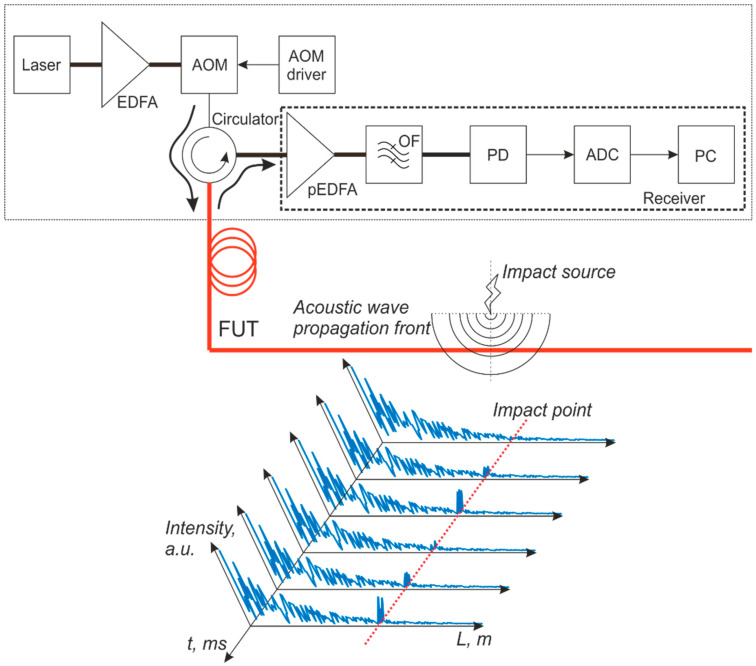
Scheme of a sensor based on a φ-OTDR.

The operating principle of an incoherent φ-OTDR is based on sending a probing optical pulse into an optical fiber, acting as a sensor, followed by recording the backscattered radiation interference signal. A typical scheme of such φ-OTDR is shown in Figure 4.

Radiation from an extremely stable (amplitude and frequency) narrow-band continuous-wave laser source is increased to the required power level, depending on the monitored line length, using an EDFA. Starting with stable laser radiation is important because it provides for high-precision system operation. Then, a train of probing optical pulses is formed from continuous radiation using an AOM controlled by its driver in a square-wave periodic modulation. The duration and duty cycle of the AOM driving pulses, and hence of the produced “on–off” optical pulses, are chosen depending on the desired spatial resolution to be obtained in the monitored region. After the AOM, there is an optical circulator, which passes ongoing radiation from the AOM into the FUT and also passes the backscattered radiation from the FUT into the receiving part of the system. A backreflected wave can be formed in an optical fiber due to different causes: reflections and Mie scattering, from surface changes and defects comparable in size to the radiation wavelength, or due to Rayleigh scattering from fiber inhomogeneities and defects much smaller than the wavelength. The last light scattering occurs homogeneously in all directions (isotropic scattering), and, in particular, it is produced in any optical fiber at the various inhomogeneity points of the refractive index, which are always present in a real optical fiber [53]. At Rayleigh scattering centers, i.e., fiber inhomogeneities whose size is small compared to the wavelength, radiation is scattered in all directions, thus including part of the radiation scattered backward. The Rayleigh backscattering coefficient is defined as the fraction of the forward wave power scattered back into the fundamental mode of the fiber from a section with a length equal to the half-width of the light pulse in the fiber [53]. The distribution of Rayleigh scattering centers is uniform along the optical fiber and originates in the fiber production process itself. For φ-OTDR, in which the laser coherence length is much longer than the scanning pulse spatial width, the amplitudes of backscattered waves are summed coherently, i.e., taking into account their phases. This leads to a random jagged reflectogram that remains stable in the absence of external (acting on the FUT) and internal (laser source, interferometer, photodetectors) changes to the system. Weak backscattered radiation enters the receiving part of the OTDR through the circulator. It is preamplified by another EDFA (pEDFA), after which there is a narrow-band optical filter (OF) filtering out the power of amplified spontaneous emission (ASE) by the EDFA at spurious wavelengths, i.e., different from the laser wavelength and outside of the passband optical filter. Finally, the filtered radiation enters the photodetector (PD), is digitized by a high-speed ADC (>100 MHz sampling frequency), and undergoes computer processing.

The resulting interference signal comes from the spatial resolution section along the fiber, where backscattered Rayleigh radiation is formed as a result of the addition of backscattered waves from all inhomogeneities within the half-width spatial length of the probing pulse along the fiber. Each backscattered wave has a random amplitude with a Gaussian probability density distribution, and the change in the phase of each wave is due to the physical displacement of the corresponding scattering center: this provides a nonlinear contribution to the final signal registered by the system [23]. Thus, it is quite difficult to obtain an obvious pattern between the introduced disturbance acting on the sensing fiber and the corresponding change in the detected signal. Such measurements can be considered as qualitative: they actually show the presence of an impact, but starting from a distorted phase signal, it is not possible to obtain an accurate measurement of the interaction with the fiber sensor.

One of the ways to increase the stability of a phase unwrapping system is to increase the SNR of the signal. For this task, one can use reflection instead of scattering the sensing pulse optical radiation and realize distributed reflections along the fiber path. In recent years, many works have been devoted to φ-OTDR based on weak fiber Bragg gratings (WFBG) [24,54,55,56,57]. The basic difference between distributed reflection sensors and distributed backscattering sensors, is that when using WFBGs, the received signal is not coming from the interference of backscattered radiation from millions of Rayleigh scattering centers, uniformly distributed along the whole fiber length. Instead, the received signal comes from the reflections arising from pairs of adjacent WFBGs, pre-inscribed in the fiber, forming an array of spatially localized interferometers displaced along the FUT. In this case, with any wide enough deformations (periodic or linear) of the fiber between a pair of WFBGs, the interferometric signal amplitude will change from its minimum to its maximum value according to the harmonic changes in the optical phase of the backreflected optical wave.

As noted earlier, the error in phase unwrapping of such systems depends on the SNR, RIN, and phase noise of the laser source, the photodetector noise, errors due to temperature gradients, and the overall vibration immunity of the system, and in particular the MZI. Various sources of noise in the φ-OTDR [58], such as photocurrent shot noise, thermal noise of electronics, thermodynamic noise of the probed fiber, and various technical noises associated with the laser source, contribute to various components for different frequencies. For example, thermodynamic noise begins to preponderate over shot noise at frequencies of several tens of hertz and below, while at higher frequencies, its contribution is less significant for single-mode fibers operating at room temperature. Due to random fluctuations in the fiber temperature, additional low-frequency noise may arise due to the random transition of the system into the intensity-blind spots [59]. The impact of RIN and laser phase noise on the system quality can be greatly reduced, in the current conditions of technological advances, by using a highly stable laser with a narrow oscillating bandwidth (e.g., in the order of 100 Hz laser linewidth). Unfortunately, the same cannot be said about a passive element in the receiving part of the system: A recording interferometer that undergoes temperature and vibration fluctuations. A possible way to reduce the last problem is by installing the recording interferometer inside an isolating box, which will not be 100% effective, and the more inert such a box is, the more cumbersome it will be. Also, such a device is something not standard in practical φ-OTDR systems.

## 3. Experiment

**Figure 5:**
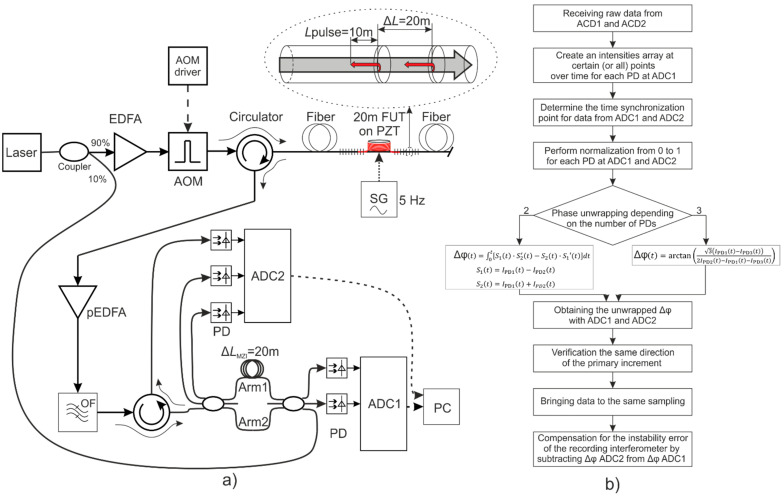
Scheme (**a**) and algorithm (**b**) of the experiment.

The scheme that we wanted to improve in our experiment is presented in Figure 5a and is a modification of the basic one for incoherent φ-OTDR with fixed time delay at the receiver (given by the MZI arms unbalance), described in Figure 2b. An NKT Photonics Koheras BASIK (with 100 Hz linewidth with 120 μs integration time) was used as laser source, the AOM (max pulse repetition rate = 1 kHz, contrast ratio = 69 dB) generated pulses 100 ns wide (excluding fronts) with repetition rate 1 kHz, IPG Photonics devices were used as EDFAs, and they have the following parameters of noise factor: EDFA 5.5 dB and pre-EDFA 4.6 dB. In the receiving part, unlike the conventional φ-OTDR scheme, our setup additionally has an unequal-arms 3 × 3 MZI, in which the sensing and reference fibers differ by a length of Δ*L*_MZI_ = 20 m (gauge length). Two outputs of the recording interferometer, in the direct path, are connected to two photodetectors of the φ-OTDR, whose outputs are then sent to the analog to digital converters marked as ADC1 (sampling frequency: 50 MHz, resolution: 16 bit). Up to this point, the detection is very similar to the one conventionally used, but it is sensitive to the MZI and laser source instabilities. In the reverse path of the MZI, we propose that all three outputs of the MZI (one through an additional circulator) are connected to the other three photodetectors (InGaAs-PIN, DC bandwidth (@ −3 dB) is 200 MHz, rise/fall time (10–90%) is 1.8 ns, min NEP (@ 10 MHz) 5.2 pW/√Hz (@ 1550 nm), whose outputs are A/D converted by the block ADC2 (max sampling frequency: 10 MHz (operating 100 kHz), resolution: 14 bit, bandwidth up to 1.2 MHz, typical SNR 73 dB). These three additional photodetectors record a fraction of the direct laser radiation (the same laser providing the optical pulses for the FUT sensing) passing through the MZI (the same MZI providing the recording of the φ-OTDR signal). In this way, a fraction of the original laser radiation, not backscattered from the FUT and hence unaffected by the sensing fiber variations under measurement, goes through the transmission of the 3 × 3 coupler MZI in the reverse direction. This optical signal is used to analyze the wavelength instability of the laser source as well as the instability of the 3 × 3 MZI, both of which can be compensated after this detection using mathematical processing. The method proposed here significantly improves the accuracy of the ultimate phase unwrapping results by cleaning up the useful phase changes caused by the recorded effect acting on the sensing fiber from additional and unwanted phase changes arising from the two most significant disturbance contributions in a φ-OTDR system (namely, MZI and laser source instabilities). In fact, in practical φ-OTDR systems, the two main disturbance contributions to the measured phase signal are due to the following: (i) Temperature instabilities of the recording interferometer; (ii) Instabilities of the laser source wavelength. The algorithm used in the experiment is shown in Figure 5b.

A standard single-mode fiber is used as the FUT, in which two WFBG arrays were inscribed at a distance of 20 m, and the parameters of the WFBGs are indicated in Table 1.

The fiber section between the two adjacent WFBGs is wound on a piezoceramic cylinder (PZT) to provide controlled fiber perturbations and optical phase changes in the sensing part of the FUT. A sine voltage signal at 5 Hz is applied to the PZT, and its amplitude values are presented in Table 2, together with the amplitudes of the resulting fiber stretching (linearly related to voltage with a sensitivity of 1 μm/V).

**Figure 6:**
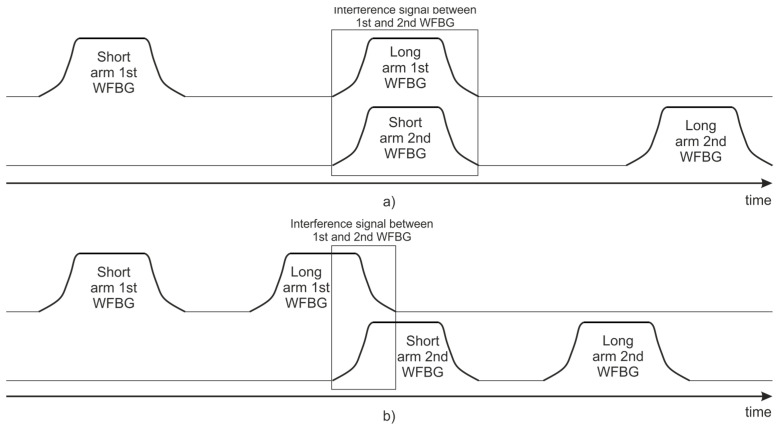
Scheme of the interference signal formation at the PD, after passing through the unbalanced MZI: (**a**) Δ*L*_MZI_ = 2Δ*L*_WFBGs_; (**b**) Δ*L*_MZI_ < 2Δ*L*_WFBGs_.

The unequal-arms MZI unbalance used in the experiment is set equal to the distance between the two adjacent WFBGs in the FUT, which is 20 m. The spatial width of the probing optical pulse in the fiber is 10 m, which is half of the distance between the WFBGs and, thus, half of the MZI arms unbalance. Therefore, at the two PDs of the direct path of the MZI, one can observe the interference of the signals from the two adjacent WFBGs inscribed along the FUT, as schematically illustrated in Figure 6.

Typically, in such incoherent φ-OTDR systems, a fiber interferometer at the detector, which is also an insulating casing, is used to provide some temperature stability (and also some insulation from vibrations) for the recording interferometer. However, in our experiment, the insulated casing is intentionally not used in order to increase the instability of the 3 × 3 MZI as produced by fluctuations in the environmental conditions and, in particular, by temperature variations. In this way, the 3 × 3 MZI can also experience parasitic disturbances from directed air flows and ambient vibrations, which are both present in the case of a typical installation of a φ-OTDR interrogator (receiving part of the system including the recording interferometer and optical detectors) into a server rack.

## 4. Analysis

A single reflectogram of an incoherent φ-OTDR system is the result of recording, after digitizing the signal, of one probe pulse arriving at the PD. A set of more reflectograms arranged on a special bidimensional graph is called a waterfall: in this graph, the distance from the beginning of the fiber line is plotted along the X-axis, time is plotted along the Y-axis, and the color of the plot (in a third virtual “Z-axis”) represents the signal intensity. The waterfall is thus a 2D-fake-colors plot, showing the interference signal intensity as a function of both distance (obtained as the product of pulse velocity in the fiber and time) and time (temporal evolution at a fixed target, sensing region, or distance). Taking from the reflectogram a specific time slice along the Y-axis, at a given spatial coordinate, on the X-axis, one gets the influence signal variations in time at this given location along the sensing cable. In our analysis, we consider a single slice corresponding to the fiber section wound on the PZT since this part of the fiber is the one affected by the voltage signal applied to the PZT.

**Figure 7:**
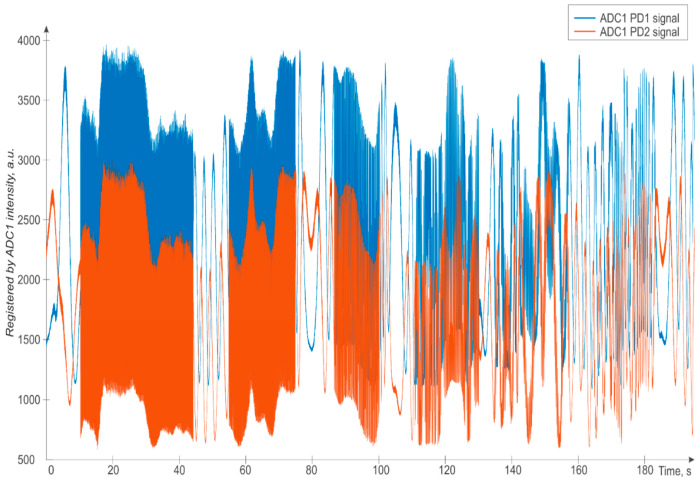
Time signal at the point of perturbation (PZT-wounded fiber region) registered by the two photodiodes in the MZI forward direction and converted by ADC1.

The time slice of data recorded by both PDs of the direct path during one of the experiments is presented in Figure 7. We can see that in some places, the signal oscillates extremely quickly—these are the time intervals where the perturbation to the FUT is applied by the piezoelectric actuator. During time intervals without impacts acting on the FUT, we can see weak opposite oscillations of the signals from the two different receivers: in fact, the two signals are shifted by 2π/3 (due to the fixed phase shift introduced by the 3 × 3 MZI), and at the moments of “crossing” through ±π they change direction. Thus, the intensities of the two signals lie within a certain range and do not grow out of such range when the perturbation has high amplitudes.

**Figure 8:**
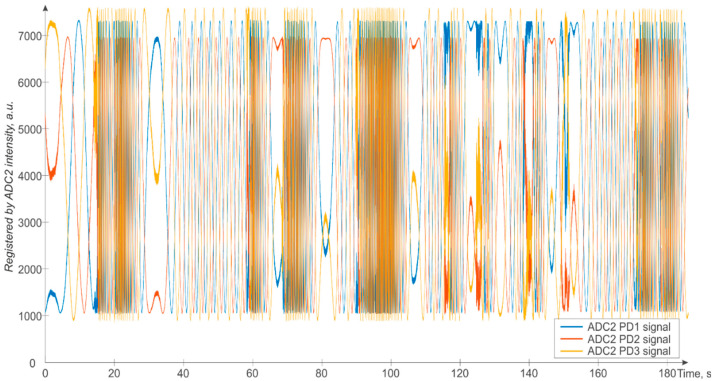
Time signal registered by ADC2.

The second group of photodetectors, which are the PDs before the converters ADC2, register the signal variations independently of the FUT variations and due to internal changes in the interferometric measurement system, which are temperature and vibration instabilities of the MZI and instability of the laser wavelength. An example of these data is shown in Figure 8. As one can see from Figure 8, the spurious/unwanted signal variations due to instabilities in the system are comparable in amplitude to the impact-driven/wanted signal variations of Figure 7. This implies that careful compensation of the system instabilities (when they cannot be reduced in advance) is mandatory to obtain a clear interpretation of the interferometric signal and its fluctuations coming from the fiber sensor and the perturbations acting on it.

As stated above, the recorded phase data always lie within the range ±π. To obtain the absolute value of the phase change, the use of phase unwrapping methods is required [60,61,62,63]. For a 3 × 3 MZI, the phase difference between adjacent outputs is $\frac{2}{3}\pi$, and having the signals from at least two of the three available photoreceivers, one can calculate the phase change, $\left(t\right)$ in the φ-OTDR sensor, by using the following formulas [51]:

(2) $$\left[\begin{array}{l} I_{PD1}\left(t\right)=I_{1}+I_{2}+2\sqrt[{\;}]{I_{1}\cdot I_{2}}\cdot \cos\left(2\frac{\pi }{\lambda }\Delta L\left(t\right)+\varphi_{0}\right)\\ I_{PD2}\left(t\right)=I_{1}+I_{2}+2\sqrt[{\;}]{I_{1}\cdot I_{2}}\cdot \cos\left(2\frac{\pi }{\lambda }\Delta L\left(t\right)+\varphi_{0}+2\pi /3\right)\\ I_{PD3}\left(t\right)=I_{1}+I_{2}+2\sqrt[{\;}]{I_{1}\cdot I_{2}}\cdot \cos\left(2\frac{\pi }{\lambda }\Delta L\left(t\right)+\varphi_{0}-2\pi /3\right) \end{array}\right.$$

(3) $$∆\varphi \left(t\right)=\int_{0}^{t}\;\left[S_{1}\left(t\right)\cdot S_{2}^{\prime }\left(t\right)-S_{2}\left(t\right)\cdot {S_{1}}^{\prime }\left(t\right)\right]dt$$

where

$$S_{1}\left(t\right)=I_{PD1}\left(t\right)-I_{PD2}\left(t\right)$$

$$S_{2}\left(t\right)=I_{PD1}\left(t\right)+I_{PD2}\left(t\right)$$

$I_{PDi}\left(t\right)$—photocurrent signal as detected by the *i*th PD, then sent as voltage to the ADC;

$I_{i}$—radiation intensity passing through the *i*th arm of the MZI;

$\varphi_{0}$—initial phase;

$\Delta L\left(t\right)$—applied fiber extension; and the primed variables represent the first derivatives in time.

Using the signals just from two PDs leads to a larger error in the phase reconstruction than using three PDs because the larger number of used PDs helps in noise averaging and also since the derivative operator used in the two PDs’ algorithm is more sensitive to noise. Therefore, to reduce the error of the phase unwrapping algorithm, it is preferable to use the signals coming from all three PDs (connected to the block ADC2 in Figure 5a) operating in the reverse direction of the MZI. The problem of wrong phase unwrapping when crossing the 2π value is cumulative in nature, and, as will be shown further, it can significantly affect the final result, especially during long-term measurements. As given in [51], it is possible to unwrap the phase using the three PDs amplitude signals by the following formula:

(4) $$\Delta \varphi \left(t\right)=arctan\left(\frac{\sqrt[{\;}]{3}\left(I_{PD1}\left(t\right)-I_{PD3}\left(t\right)\right)}{2I_{PD2}\left(t\right)-I_{PD1}\left(t\right)-I_{PD3}\left(t\right)}\right),$$

which does not involve signal derivatives.

It must be highlighted that the recorded phase variations on ADC1 and ADC2 have different sources. For the interference signal detected by ADC1, as one can see from (5), we have the following: (1) The result of the doubled phase difference between two WFBGs in a pair; (2) The addition of phase instability of the MZI in the receiver part, accounted in the Δ*L*_MZI_(*t*) term as instability of the interferometer unbalance, also including the instability arising from the laser source frequency/wavelength changes, accounted in the *λ*(*t*) variable term. Instead, for the signals detected by ADC2, as one can also see from (6), we have no phase contribution from the backreflected waves from the sensing fiber (and the inscribed WFBGs), and the phase variations result only from the phase instability of the MZI in the receiver part, Δ*L*_MZI_(*t*), including its instability arising from the laser source frequency changes, *λ*(*t*). These two signals, the first one from ADC1 used for the phase measurement including the instabilities and the second one with a phase dependence only on the instabilities, can be described by the following equations:

(5) $$I_{ADC1}\left(t\right)\sim cos\left(\frac{4\pi L_{WFBGi}\left(t\right)}{\lambda \left(t\right)}+\frac{2\pi L_{MZI}\left(t\right)\left(1+CTE\cdot T\left(t\right)\right)}{\lambda \left(t\right)}\right),$$

(6) $$I_{ADC2}\left(t\right)\sim cos\left(\frac{2\pi L_{MZI}\left(t\right)\left(1+CTE\cdot T\left(t\right)\right)}{\lambda \left(t\right)}\right),$$

where $L_{WFBGi}$ is the fiber extension between the *i*th and (*i* + 1)th WFBGs and *CTE* is the coefficient of thermal expansion of the optical fiber, thus determining the MZI length variation due to heating or cooling by a value Δ*T* from the initial temperature.

The time dependence of $\lambda \left(t\right)$, as already stated, describes the instability of the laser wavelength. For the practical linewidths of the good and best narrow linewidths laser sources available for φ-OTDR systems, this component to the detected phase instability can be ignored (since it produces a much smaller effect on the phase than the one resulting from the instability of the MZI) and hence the natural wavelength instability of the laser can be neglected, thus assuming a constant laser wavelength in Equation (5).

The observed time dependence of Δ*L*_MZI_(*t*) shows that this difference in MZI arms is mainly unstable due to mechanical vibrations of the interferometer fibers. Therefore, we consider this parameter as the main source of measurement inaccuracy for low-frequency signals. To eliminate the phase error coming from this source, we can use the signal from ADC2 (including just MZI and laser instabilities) in combination with the measurement signal from ADC1 (including the sensing fiber signal as well as MZI and laser instabilities).

As the same laser source and the same interferometer are used at the same time to generate all the phase signals in the system, we can use data from ADC2 to compensate for the error from MZI and laser instability on the data from ADC1.

For comparison, applying the Formulas (3) and (4) to the recorded data, we obtain the following phaseshift signals:

– Phase unwrapped by 2 PDs from the φ-OTDR’s signal, which is affected by both internal factors (instability of the laser frequency, temperature and vibration influences on the MZI, etc.) and external factors (temperature changes in the sensor cable, direct signal from the impact of the PZT to fiber);
– Phase unwrapped by 3 PDs from the signal on the photodetectors connected to ADC2, which is affected only by the internal factors;
– Phase from the signal at the photodetectors connected to ADC2 unwrapped for each pair;
– Difference between the phase signal from the φ-OTDR and the phase signal on the photodetectors connected to ADC2.

**Figure 9:**
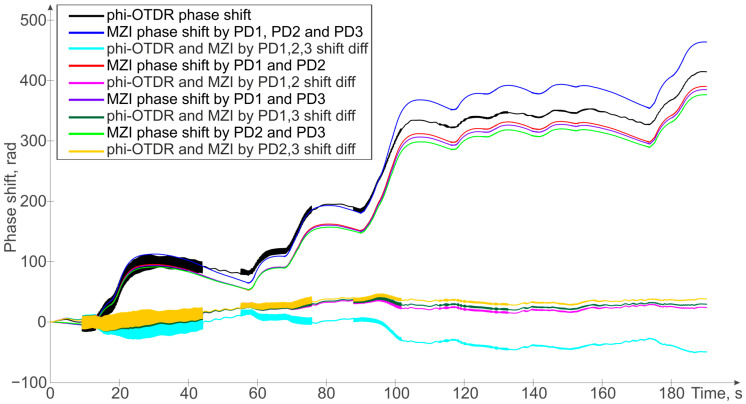
Unwrapped signals from ADC1 and ADC2, differences between the signal from the φ-OTDR and the signals on the photodetectors connected to ADC2.

The obtained results, showing different phaseshift signals, are provided in Figure 9.

As mentioned earlier, for the phaseshift formula using two PDs, the phase unwrapping error is quite higher than for the phaseshifts obtained using three PDs. Even using different pairs of PDs from the three available ones in the direct path (from the laser to two photodetectors of the φ-OTDR as mentioned above), it is clear that the final result for them will be slightly different, which will lead to an increase in error when increasing the time of the measurement (see Figure 9). Thus, to increase the accuracy of measurements, three PDs should also be located in the MZI reverse path in the receiving part of the φ-OTDR.

**Figure 10:**
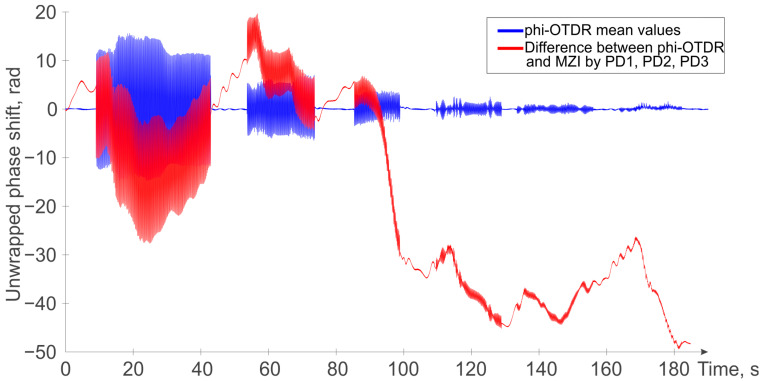
Comparison of the signal obtained by low-pass filtering (blue trace) and the signal obtained by the proposed method (red trace).

Additionally, it is worth noting that this method of compensating for instability is appropriate and works very well when it is necessary to monitor slow temperature changes in the area monitored by the FUT. For example, a comparison of the proposed method with the result of a simple low-pass filter (φ-OTDR mean values) is shown in Figure 10. As one can see, all the effects from the PZT interactions are clearly visible in both cases. However, while for low amplitudes of impact, the bandpass filter shows even clearer results (being unaffected by temperature variations), this simple filtering, unfortunately, rules out completely all slow processes acting on the phase shift signal, like temperature changes, which in certain cases may be of particular interest. In the presence of moderate and medium–high amplitudes of impacts, the proposed and more sophisticated filtering by using more photodiodes in both direct and reverse paths of the interferometer, provides for efficient disturbance canceling (from MZI and laser instabilities) while still allowing good φ-OTDR sensitivity to temperature changes and other slow perturbations acting on the sensing fiber.

## 5. Conclusions

The paper examines the importance and influence of the recording interferometer instability (when the laser instability contribution is negligible) on the result of phase measurements in φ-OTDR schemes. A compensation scheme based on an MZI formed by 3 × 3 couplers and used in both the direct and reverse paths is proposed and applied to significantly reduce the effects of the interferometer instability on the φ-OTDR phase signal. A comparison between signals with or without such compensation is presented. It is shown that using different pairs or all three available photodetectors in the MZI reverse path retains different errors of phase unwrapping. In particular, the use of all three photodetectors allows much better unwrapped-phase reconstruction, with insensitivity to the MZI mechanical and thermal instabilities, as well as potentially to laser source wavelength instability when non-negligible in the beginning.

## Figures and Tables

**Table 1:** Parameters of WFBG array.

Distance between WFBGs	20 m
Central wavelength	1550 ± 1 nm
Reflection coefficient	0.3%

**Table 2:** Parameters of the piezoceramic cylinder and applied voltage amplitudes.

Shape of Impact Interaction	Sinewave at 5 Hz Frequency					
Applied voltage, V	5	2	1	0.5	0.2	0.1
Resulting stretching magnitude, nm	5000	2000	1000	500	200	100

## Data Availability

The data presented in this study are available on request from the corresponding author.
